# Author Correction: Intrathecal delivery of adipose-derived mesenchymal stem cells in traumatic spinal cord injury: Phase I trial

**DOI:** 10.1038/s41467-024-48979-7

**Published:** 2024-06-05

**Authors:** Mohamad Bydon, Wenchun Qu, F. M. Moinuddin, Christine L. Hunt, Kristin L. Garlanger, Ronald K. Reeves, Anthony J. Windebank, Kristin D. Zhao, Ryan Jarrah, Brandon C. Trammell, Sally El Sammak, Giorgos D. Michalopoulos, Konstantinos Katsos, Stephen P. Graepel, Kimberly L. Seidel-Miller, Lisa A. Beck, Ruple S. Laughlin, Allan B. Dietz

**Affiliations:** 1 Neuro-Informatics Laboratory, Mayo Clinic, Rochester, MN USA; 2 Department of Neurological Surgery, Mayo Clinic, Rochester, MN USA; 3 Physical Medicine and Rehabilitation, Mayo Clinic, Jacksonville, FL USA; 4 Department of Pain Medicine, Mayo Clinic, Jacksonville, FL USA; 5 Physical Medicine and Rehabilitation, Mayo Clinic, Rochester, MN USA; 6 Department of Neurology, Mayo Clinic, Rochester, MN USA; 7 Department of Laboratory Medicine and Pathology, Mayo Clinic, Rochester, MN USA

**Keywords:** Spinal cord diseases, Stem-cell therapies, Spinal cord injury

Correction to: *Nature Communications* 10.1038/s41467-024-46259-y, published online 01 April 2024

The original version of this Article contained an error in the text in the first paragraph of the Results, which incorrectly read ‘The average duration between injury and AD-MSC injection was 11 months (range of 8–22)’. The correct version replaces this sentence with ‘The average duration between injury and AD-MSC injection was 12 months (range of 7–22)’. This has been corrected in both the PDF and HTML version of the Article.

The original version of this Article contained an error in Table 1, in which Patient 1 was incorrectly reported to have a time between injury and injection as 22 months. The correct version states the time between injury and injection as 11 months.

The correct version of Table 1 is:

**Table 1:** 

Patient #	Injury Level	Race	AIS at Injury	AIS at Enrollment	AIS at Injection	AIS at Week 96	Component driving AIS change after injection	Time between injury and enrollment	Time between injury and injection
Patient 1	Cervical C5-C6	White	A	C	C	D	More than half of the key muscles below the level of injury graded 3 or better	10 months	11 months
Patient 2	Thoracic T11-T12	White	A	A	A	A		10 months	11 months
Patient 3	Cervical C8	White	B	B	B	C	Voluntary anal contraction	8 months	10 months
Patient 4	Thoracic T12	Hispanic/ Latino	A	B	C	C		10 months	14 months
Patient 5	Thoracic T10	White	A	A	A	C	Deep anal pressure and voluntary anal contraction	6 months	8 months
Patient 6	Cervical C6	White	A	A	A	B	Deep anal pressure	11 months	13 months
Patient 7	Thoracic T11	Black	A	A	A	C	Deep anal pressure and motor function more than three levels below the motor level	9 months	10 months
Patient 8	Cervical C4	Asian	A	A	A	A		2 months	7 months
Patient 9	Cervical C7	White	B	C	C	D	More than half of the key muscles below the level of injury graded 3 or better	10 months	22 months
Patient 10	Cervical C5-C6	White	A	B	B	C	Voluntary anal contraction	12 months	14 months

Which replaces the previous incorrect version:

**Table 2:** 

Patient #	Injury Level	Race	AIS at Injury	AIS at Enrollment	AIS at Injection	AIS at Week 96	Component driving AIS change after injection	Time between injury and enrollment	Time between injury and injection
Patient 1	Cervical C5-C6	White	A	C	C	D	More than half of the key muscles below the level of injury graded 3 or better	10 months	22 months
Patient 2	Thoracic T11-T12	White	A	A	A	A		10 months	11 months
Patient 3	Cervical C8	White	B	B	B	C	Voluntary anal contraction	8 months	10 months
Patient 4	Thoracic T12	Hispanic/ Latino	A	B	C	C		10 months	14 months
Patient 5	Thoracic T10	White	A	A	A	C	Deep anal pressure and voluntary anal contraction	6 months	8 months
Patient 6	Cervical C6	White	A	A	A	B	Deep anal pressure	11 months	13 months
Patient 7	Thoracic T11	Black	A	A	A	C	Deep anal pressure and motor function more than three levels below the motor level	9 months	10 months
Patient 8	Cervical C4	Asian	A	A	A	A		2 months	7 months
Patient 9	Cervical C7	White	B	C	C	D	More than half of the key muscles below the level of injury graded 3 or better	10 months	22 months
Patient 10	Cervical C5-C6	White	A	B	B	C	Voluntary anal contraction	12 months	14 months

