# Supplementary material for: Intrathecal delivery of adipose-derived mesenchymal stem cells in traumatic spinal cord injury: Phase I trial
Source: Nat Commun. 2024 Apr 1;15:2201. doi: 10.1038/s41467-024-46259-y (PMC10984970; doi:10.1038/s41467-024-46259-y)

Supplementary Table 1: International Standards for Neurological Classification of Spinal Cord Injury (ISNCSCI) changes.

International Standards for Neurological Classification of Spinal Cord Injury (ISNCSCI) Motor, Sensory – Pin Prick, and Sensory – Light Touch score changes from baseline at weeks 4, 24, 48, and 96.

Patient #	Total Motor Change from Baseline				Total Pin Prick Change from Baseline				Total Light Touch Change from Baseline			
	Week 4	Week 24	Week 48	Week 96	Week 4	Week 24	Week 48	Week 96	Week 4	Week 24	Week 48	Week 96
Patient 1	13	18	21	25	18	25	22	26	10	17	25	43
Patient 2	0	1	1	1	0	1	-4	0	1	1	-3	1
Patient 3	1	-5	-6	1	23	14	19	29	19	10	5	11
Patient 4	5	4	2	0	10	7	-9	-3	-1	0	-1	-6
Patient 5	-1	-3	-2	-3	-4	-2	-2	-4	-3	-2	-3	-4
Patient 6	-1	2	-1	1	9	6	7	6	8	5	2	3
Patient 7	0	0	0	2	0	-1	0	1	0	0	3	0
Patient 8	-1	-2	-1	-2	4	2	5	1	2	2	0	-3
Patient 9	27	24	34	26	1	-8	11	-11	-7	-9	-4	-13
Patient 10	4	8	11	12	5	-1	-6	3	4	-3	-6	7

Note - The values are derived by summing the scores in each domain (Motor, Pin Prick, Light Touch) for the upper and lower extremities. Subsequently, the total baseline score is subtracted from the corresponding follow-up score for each respective domain.

Supplementary Table 2: Number of sensory and motor levels improved at final follow-up.
Number of International Standards for Neurological Classification of Spinal Cord Injury (ISNCSCI) muscle function levels and dermatomes improved at the final follow-up for Motor, Sensory – Pin Prick, and Sensory – Light Touch.

Patient #	Motor Change Improvement						Pin Prick Improvement			Light Touch Improvement		
	Number of spinal levels improved	Spinal levels improved by 1	Spinal levels improved by 2	Spinal levels improved by 3	Spinal levels improved by 4	Spinal levels improved by 5	Number of spinal levels improved	Spinal levels improved by 1	Spinal levels improved by 2	Number of spinal levels improved	Spinal levels improved by 1	Spinal levels improved by 2
Patient 1	19	13	6				24	22	2	40	38	2
Patient 2	1	1								1	1	
Patient 3	1	1					28	27	1	11	11	
Patient 4							3	3		1	1	
Patient 5												
Patient 6	1	1					5	1	4	5	4	1
Patient 7	2	2					1	1				
Patient 8							2	1	1	2	1	1
Patient 9	12	5	2	4		1	2	2		3	3	
Patient 10	8	5	2	1			2	1	1	7	7	

Note - The values represent the number of levels that improved at the final follow-up compared to baseline.

Supplementary Figure 1: Patient 1 Dermatomal body map.

Dermatomal body map representing International Standards for Neurological Classification of Spinal Cord Injury (ISNCSCI) scores at baseline and final follow-up (96 weeks) for Patient 1.

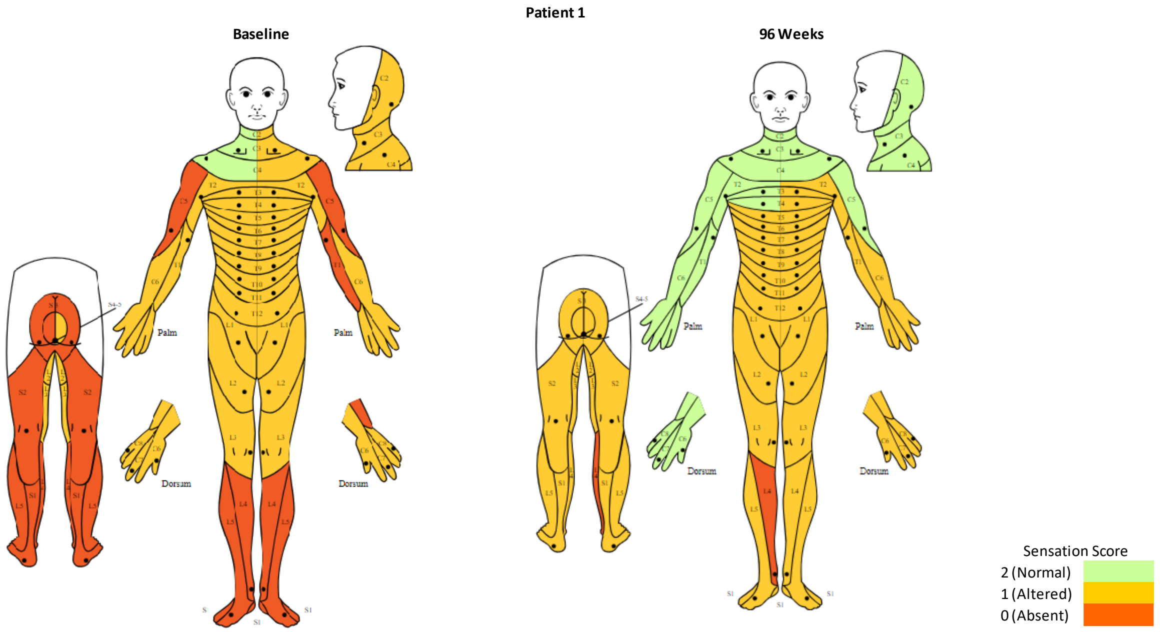

Supplementary Figure 2: Patient 2 Dermatomal body map.

Dermatomal body map representing International Standards for Neurological Classification of Spinal Cord Injury (ISNCSCI) scores at baseline and final follow-up (96 weeks) for Patient 2.

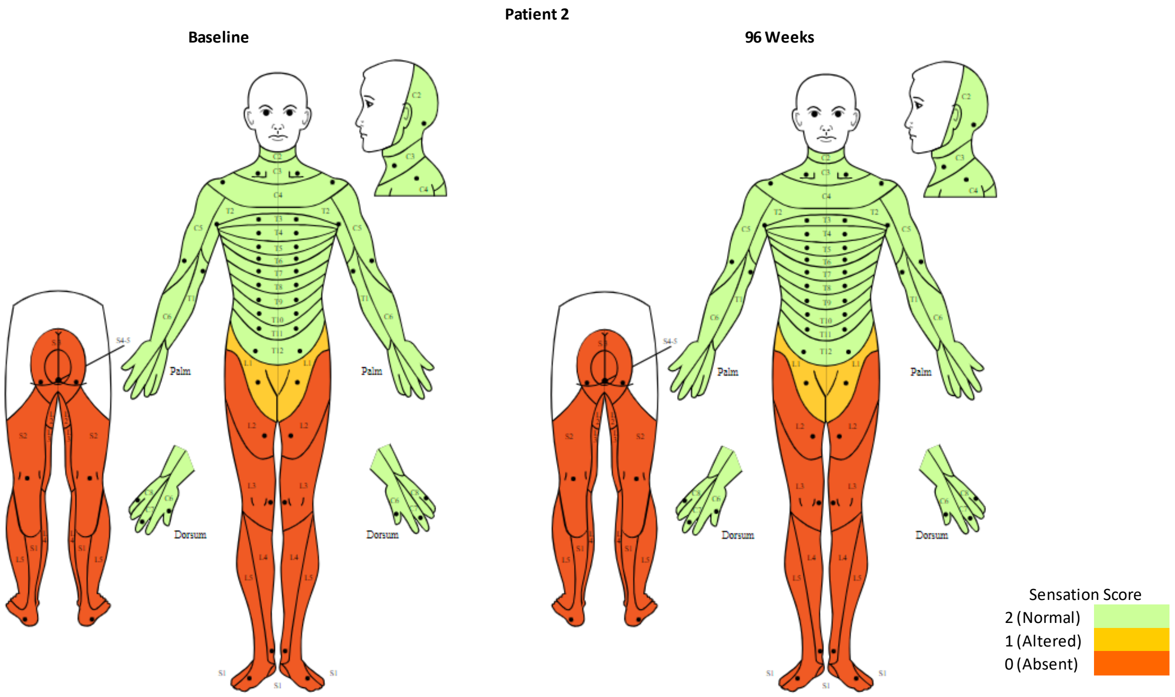

Supplementary Figure 3: Patient 3 Dermatomal body map.

Dermatomal body map representing International Standards for Neurological Classification of Spinal Cord Injury (ISNCSCI) scores at baseline and final follow-up (96 weeks) for Patient 3.

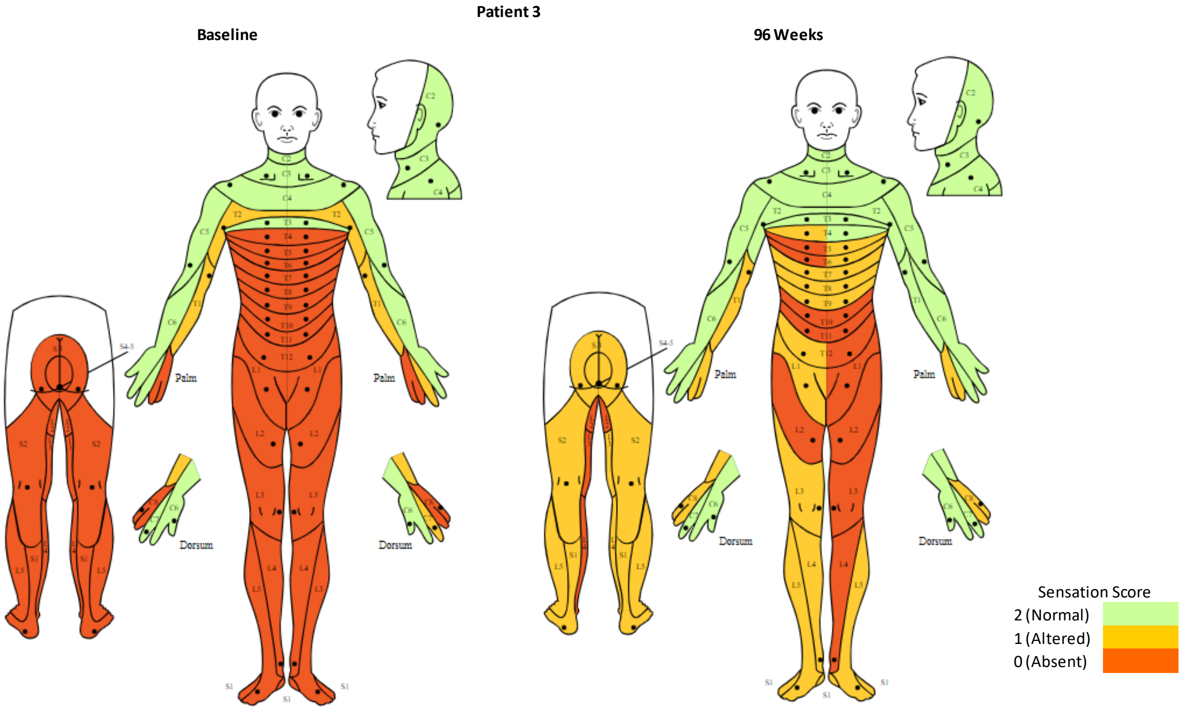

Supplementary Figure 4: Patient 4 Dermatomal body map.

Dermatomal body map representing International Standards for Neurological Classification of Spinal Cord Injury (ISNCSCI) scores at baseline and final follow-up (96 weeks) for Patient 4.

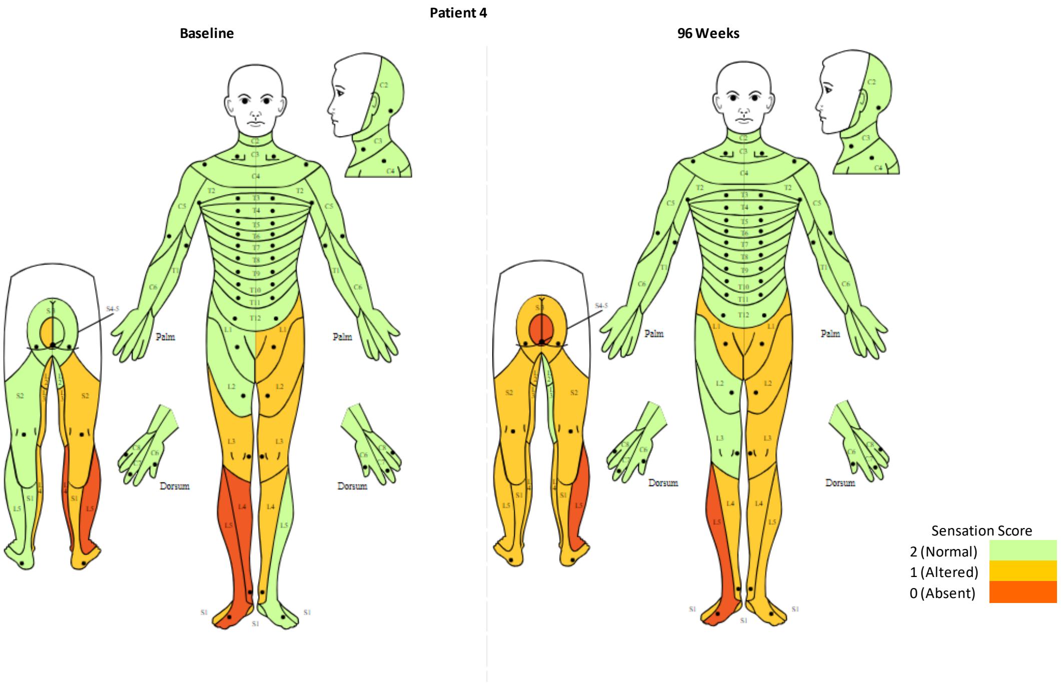

Supplementary Figure 5: Patient 5 Dermatomal body map.

Dermatomal body map representing International Standards for Neurological Classification of Spinal Cord Injury (ISNCSCI) scores at baseline and final follow-up (96 weeks) for Patient 5.

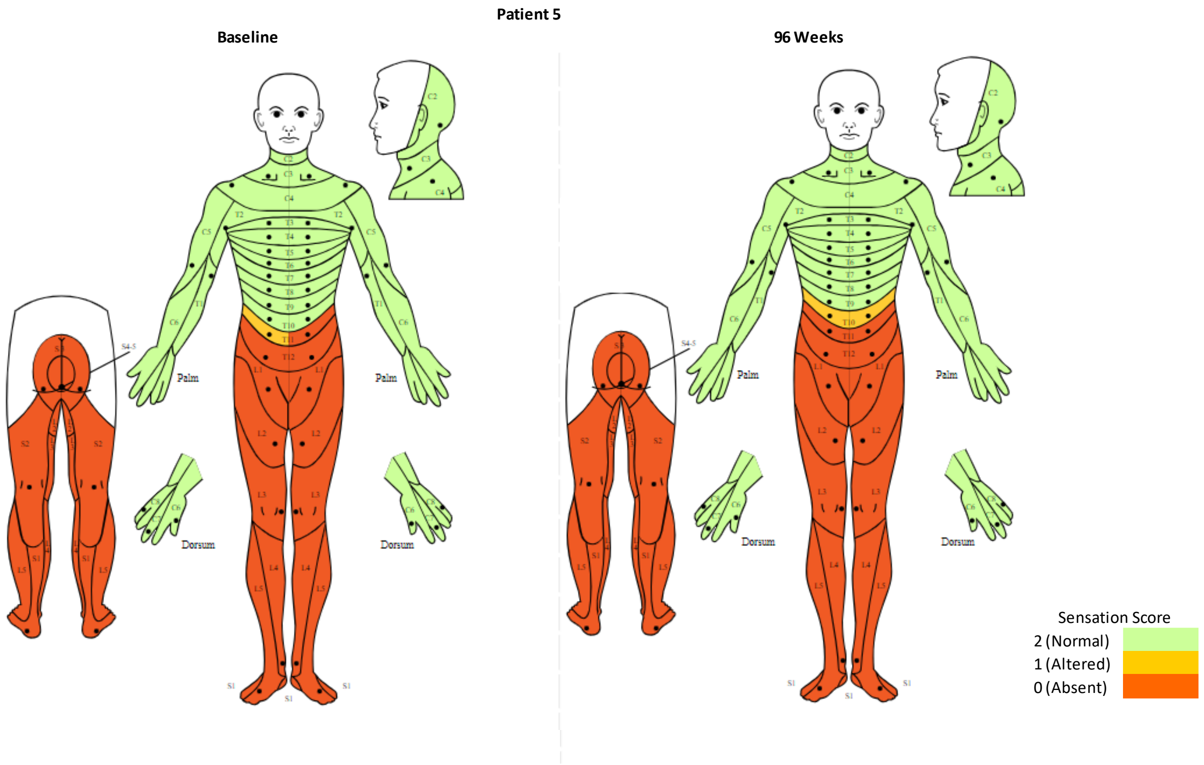

Supplementary Figure 6: Patient 6 Dermatomal body map.

Dermatomal body map representing International Standards for Neurological Classification of Spinal Cord Injury (ISNCSCI) scores at baseline and final follow-up (96 weeks) for Patient 6.

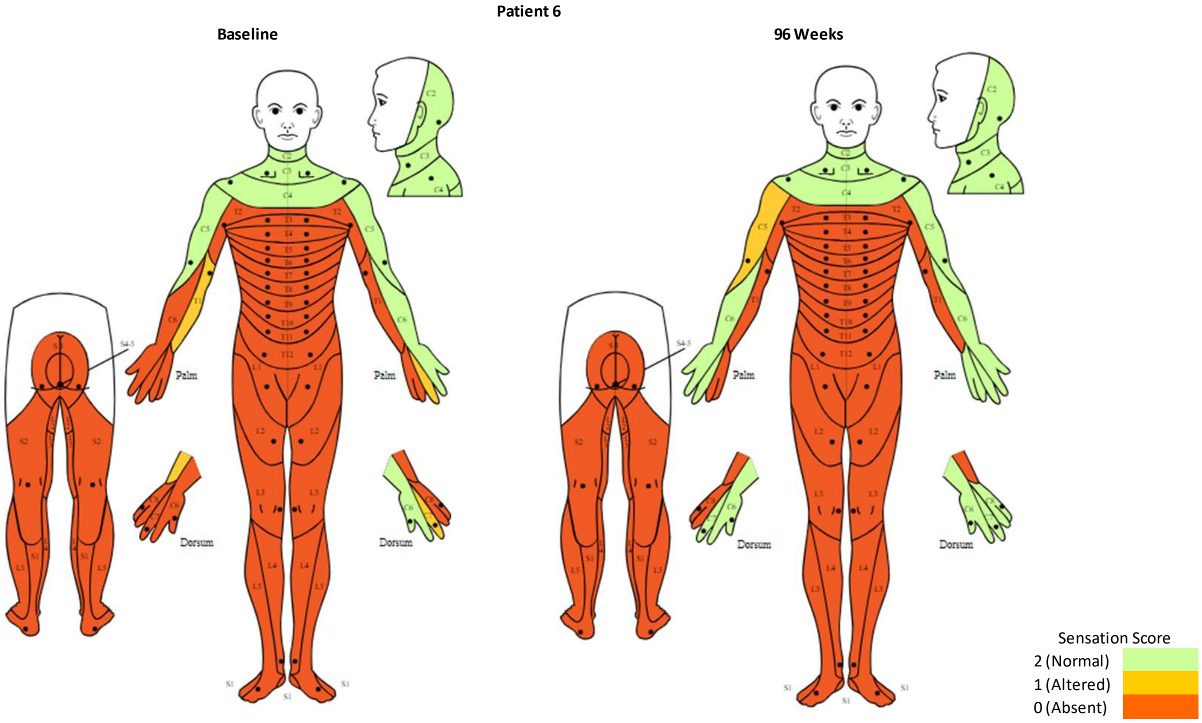

Supplementary Figure 7: Patient 7 Dermatomal body map.

Dermatomal body map representing International Standards for Neurological Classification of Spinal Cord Injury (ISNCSCI) scores at baseline and final follow-up (96 weeks) for Patient 7.

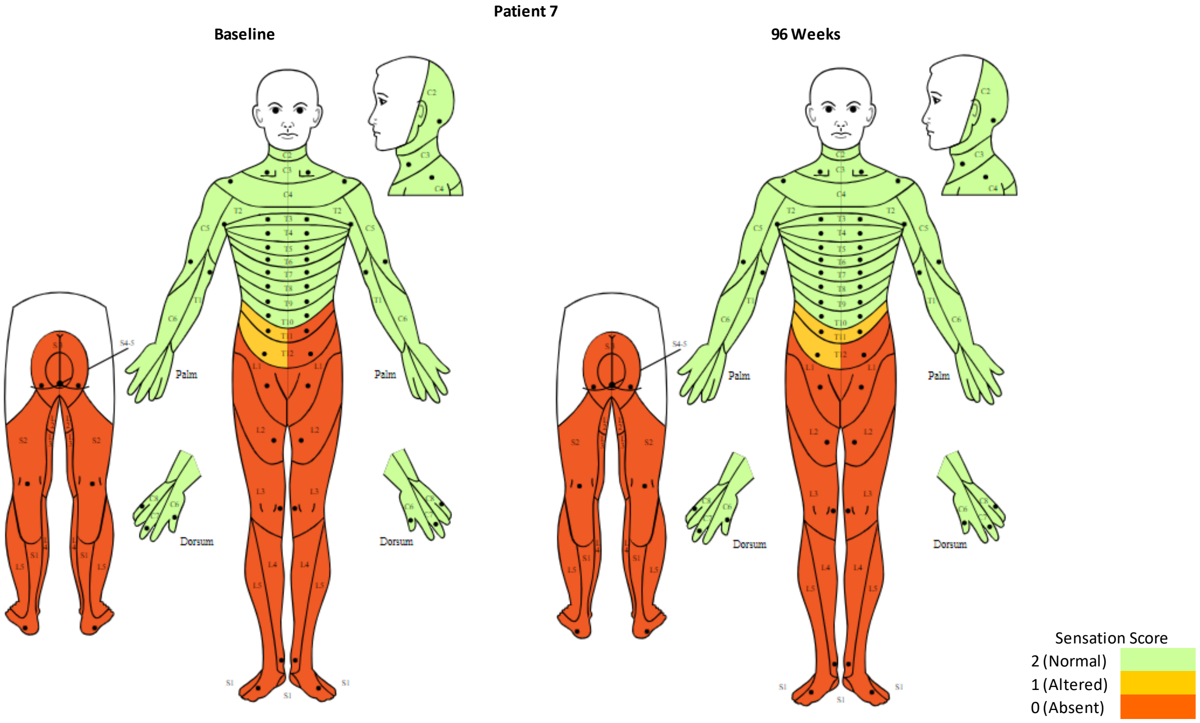

Supplementary Figure 8: Patient 8 Dermatomal body map.

Dermatomal body map representing International Standards for Neurological Classification of Spinal Cord Injury (ISNCSCI) scores at baseline and final follow-up (96 weeks) for Patient 8.

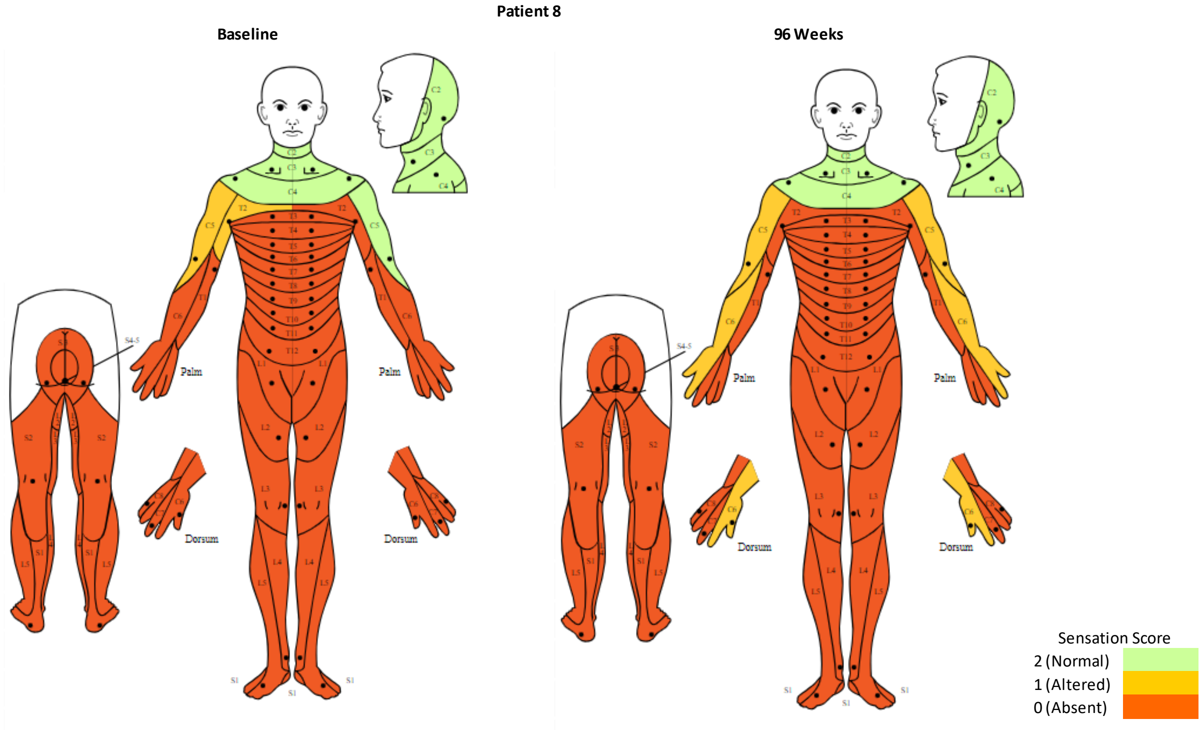

Supplementary Figure 9: Patient 9 Dermatomal body map.

Dermatomal body map representing International Standards for Neurological Classification of Spinal Cord Injury (ISNCSCI) scores at baseline and final follow-up (96 weeks) for Patient 9.

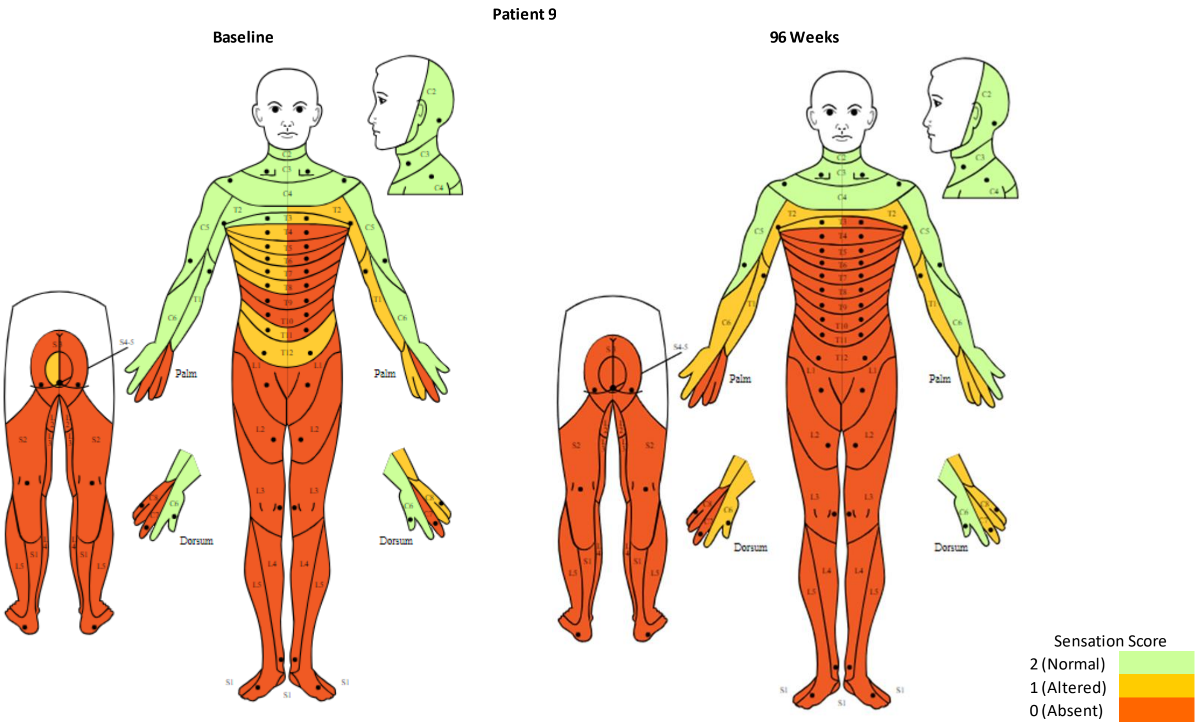

Supplementary Figure 10: Patient 10 Dermatomal body map.

Dermatomal body map representing International Standards for Neurological Classification of Spinal Cord Injury (ISNCSCI) scores at baseline and final follow-up (96 weeks) for Patient 10.

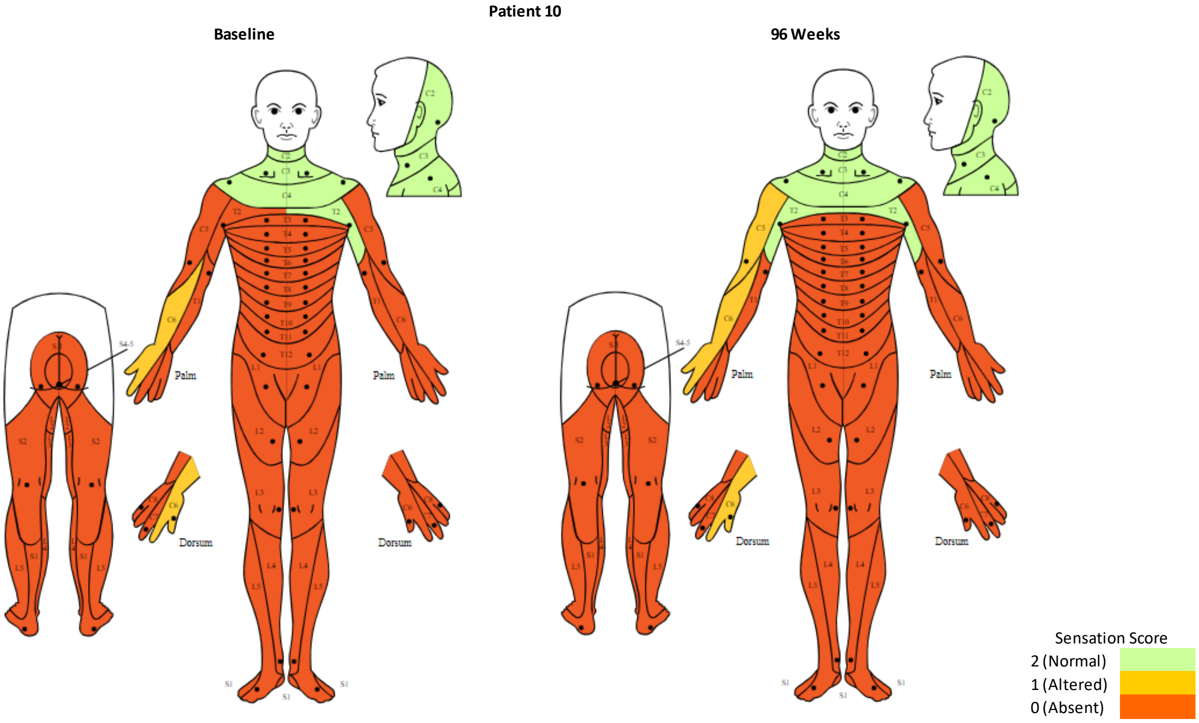

Supplement: Supplementary file 1 — Supplementary Information [file 41467_2024_46259_MOESM1_ESM.pdf]
