# Supplementary material for: Intrathecal delivery of adipose-derived mesenchymal stem cells in traumatic spinal cord injury: Phase I trial
Source: Nat Commun. 2024 Apr 1;15:2201. doi: 10.1038/s41467-024-46259-y (PMC10984970; doi:10.1038/s41467-024-46259-y)
Supplement: Supplementary file 3 — Reporting Summary [file 41467_2024_46259_MOESM3_ESM.pdf]

nature portfolio

Corresponding author(s): Mohamad Bydon

Last updated by author(s): 10/10/2023

Reporting Summary

Nature Portfolio wishes to improve the reproducibility of the work that we publish. This form provides structure for consistency and transparency in reporting. For further information on Nature Portfolio policies, see our Editorial Policies and the Editorial Policy Checklist.

Statistics

For all statistical analyses, confirm that the following items are present in the figure legend, table legend, main text, or Methods section.

n/a	Confirmed	
☐	☒	The exact sample size (*n*) for each experimental group/condition, given as a discrete number and unit of measurement
☒	☐	A statement on whether measurements were taken from distinct samples or whether the same sample was measured repeatedly
☒	☐	The statistical test(s) used AND whether they are one- or two-sided<br>*Only common tests should be described solely by name; describe more complex techniques in the Methods section.*
☒	☐	A description of all covariates tested
☒	☐	A description of any assumptions or corrections, such as tests of normality and adjustment for multiple comparisons
☒	☐	A full description of the statistical parameters including central tendency (e.g. means) or other basic estimates (e.g. regression coefficient) AND variation (e.g. standard deviation) or associated estimates of uncertainty (e.g. confidence intervals)
☒	☐	For null hypothesis testing, the test statistic (e.g. *F*, *t*, *r*) with confidence intervals, effect sizes, degrees of freedom and *P* value noted<br>*Give P values as exact values whenever suitable.*
☒	☐	For Bayesian analysis, information on the choice of priors and Markov chain Monte Carlo settings
☒	☐	For hierarchical and complex designs, identification of the appropriate level for tests and full reporting of outcomes
☒	☐	Estimates of effect sizes (e.g. Cohen's *d*, Pearson's *r*), indicating how they were calculated

Our web collection on statistics for biologists contains articles on many of the points above.

Software and code

Policy information about availability of computer code

Data collection	Data collection was performed using Microsoft Word, Microsoft Excel, Epic Electronic Health Records
Data analysis	Statistical analysis was not conducted. Findings were reported descriptively. Graphs were generated using Rstudio and Microsoft PowerPoint

For manuscripts utilizing custom algorithms or software that are central to the research but not yet described in published literature, software must be made available to editors and reviewers. We strongly encourage code deposition in a community repository (e.g. GitHub). See the Nature Portfolio guidelines for submitting code & software for further information.

Data

Policy information about availability of data

All manuscripts must include a data availability statement. This statement should provide the following information, where applicable:

- Accession codes, unique identifiers, or web links for publicly available datasets
- A description of any restrictions on data availability
- For clinical datasets or third party data, please ensure that the statement adheres to our policy

The somatosensory evoked potentials (Table 4) and the cerebrospinal fluid cytokine levels (Figure 6) are provided in the source data file with this paper. The processed data from the patients' electronic health records, pertaining to adverse events, imaging changes, and sensory/motor changes are presented in Tables 1-3, Supplementary Tables 1-2, Supplementary Images 1-10. After deidentification, individual participant data relevant to the results presented in this article, will be available upon request and will be available indefinitely. The data will be shared with investigators who provide a methodologically sound proposal and/or have obtained approval by an independent review committee. Shared data can only be used for the purposes of the approved proposal and/or individual participant data

meta-analysis. Data requests will be managed by the corresponding author via email (bydon.mohamad@mayo.edu). The study protocol is available on ClinicalTrials.gov (Identifier: NCT03308565).

Research involving human participants, their data, or biological material

Policy information about studies with human participants or human data. See also policy information about sex, gender (identity/presentation), and sexual orientation and race, ethnicity and racism.

Reporting on sex and gender	The index study included male and female participants. Information was collected via self reporting, and consent for patient level data was obtained. Subgroup analyses based on sex or gender were not conducted, as it was not deemed a relevant factor for the safety of intrathecally administered adipose-tissue derived mesenchymal stem cells for the treatment of spinal cord injury.
Reporting on race, ethnicity, or other socially relevant groupings	The index study did collected information for race and ethnicity, but did not constrain inclusion based on these factors. Information was collected via self reporting, and consent for patient level data was obtained. Subgroup analyses based on race or ethnicity were not conducted, as it was not deemed a relevant factor for the safety of intrathecally administered adipose-tissue derived mesenchymal stem cells for the treatment of spinal cord injury.
Population characteristics	Male or female aged 18 years and older.<br>AIS grade A or B of spinal cord injury (SCI)<br>SCI must be traumatic, blunt/non-penetrating in nature and not degenerative<br>SCI must be within two weeks and up to 1 year after the event
Recruitment	The study considered patients for inclusion who were either referred by medical professionals or chose to self-refer. Participants were able to find the study through https://clinicaltrials.gov/ or other publicly available spinal cord injury trial finders (e.g. https://scitrials.org/triallist). Due to the nature of the enrollment, the sample included in the study is subject to selection bias. The study included patients who were motivated and were able to engage in treatment at a specialized center, which may not necessarily be true for the average spinal cord injury patient. These limitations are discussed in greater detail in the manuscript.
Ethics oversight	This trial was reviewed and allowed to proceed by the United States Food and Drug Administration (ClinicalTrials.gov Identifier: NCT03308565) and the Mayo Clinic Institutional Review Board (IRB no. 17-004621).

Note that full information on the approval of the study protocol must also be provided in the manuscript.

Field-specific reporting

Please select the one below that is the best fit for your research. If you are not sure, read the appropriate sections before making your selection.

☒ Life sciences ☐ Behavioural & social sciences ☐ Ecological, evolutionary & environmental sciences

For a reference copy of the document with all sections, see nature.com/documents/nr-reporting-summary-flat.pdf

Life sciences study design

All studies must disclose on these points even when the disclosure is negative.

Sample size	The study included 10 patients with spinal cord injury. Sample size was determined based on clinical considerations, previous Phase I studies utilizing the same cellular product and the clinical expertise of the primary investigator.<br><br>(Staff NP, Madigan NN, Morris J, et al. Safety of intrathecal autologous adipose-derived mesenchymal stromal cells in patients with ALS. Neurology. 2016;87(21):2230-2234)
Data exclusions	No data were excluded from the analyses
Replication	The index study is a phase I trial, assessing efficacy of adipose derived mesenchymal stem cells in spinal cord injury. Replication of the results was not feasible given the clinical nature of the study. All 10 patients were studied for the safety of intrathecal stem cell injection for spinal cord injury.
Randomization	The index study is a phase I trial, assessing efficacy of adipose derived mesenchymal stem cells in spinal cord injury. All patients received intrathecal administration of stem cells and were not radnomized, as the nature of the study is not a comparative one.
Blinding	The index study is a single arm phase I trial, assessing efficacy of adipose derived mesenchymal stem cells in spinal cord injury. All patients received the same intervention (intrathecal administration of stem cells). Blinding was not conducted as there was no control/comparison arm included in the study.

Reporting for specific materials, systems and methods

We require information from authors about some types of materials, experimental systems and methods used in many studies. Here, indicate whether each material, system or method listed is relevant to your study. If you are not sure if a list item applies to your research, read the appropriate section before selecting a response.

Materials & experimental systems

n/a	Involved in the study
☒	☐ Antibodies
☒	☐ Eukaryotic cell lines
☒	☐ Palaeontology and archaeology
☒	☐ Animals and other organisms
☐	☒ Clinical data
☒	☐ Dual use research of concern
☒	☐ Plants

Methods

n/a	Involved in the study
☒	☐ ChIP-seq
☒	☐ Flow cytometry
☒	☐ MRI-based neuroimaging

Clinical data

Policy information about clinical studies

All manuscripts should comply with the ICMJEguidelines for publication of clinical research and a completedCONSORT checklist must be included with all submissions.

Clinical trial registration	NCT03308565
Study protocol	The study protocol can be accessed at clinicaltrials.gov (https://clinicaltrials.gov/study/NCT03308565#more-information)
Data collection	The study enrolled patients and collected data from 2017-12-05 to 2021-10-11. Data collection was conducted in person at regular follow-ups at the quaternary academic center conducting the study. When in-person follow-ups where not possible, telephone consultations were conducted.
Outcomes	The primary endpoint of this study was the safety profile of intrathecally administered adipose derived mesenchymal stem cells, as reflected via the nature, incidence, and severity of any AEs. Adverse events were defined as any untoward or undesirable medical occurrence in the form of signs, symptoms, abnormal findings, or diseases that emerge or worsen relative to baseline (regardless of whether the AE had a causal relationship with the study drug). Serious AEs were defined as events that involve any of the following: death, life-threatening adverse experience, new inpatient hospitalization or prolonged hospitalization, disability, or birth defect/ anomaly. The secondary endpoints for this study were changes in patients' sensory and motor scores. Neurologic level of injury and severity scores were assessed by Physical Medicine and Rehabilitation Physicians or Advanced Nurse Practitioners, which included AIS grading. Each patient was scheduled to receive a total of ten examinations after injection. The AIS grading was used to assess the sensory and motor levels of each patient. The scale has five classification levels, ranging from complete loss of neural function in the affected area (Grade A) to completely normal (Grade E). Magnetic resistance images of the spine, somatosensory evoked potentials, and CSF cytokines analyses were performed.

Plants

Seed stocks	n/a
Novel plant genotypes	n/a
Authentication	n/a
